# Evidence from a multicenter CMR registry indicates that stress CMR imaging provides highly effective risk stratification in patients suspected to have myocardial ischemia

**DOI:** 10.1186/1532-429X-16-S1-M1

**Published:** 2014-01-16

**Authors:** Amit R Patel, Kevin Steel, Caroline A Daly, Akhil Narang, Subha V Raman, Raymond Y Kwong

**Affiliations:** 1 Brigham and Women's Hospital, Boston, Massachusetts, USA; 2 University of Chicago, Chicago, Illinois, USA; 3 The Ohio State University Medical Center, Columbus, Ohio, USA; 4 Wilford Hall Medical Center, San Antonio, Texas, USA; 5 St. James Hospital, Dublin, Ireland

## Background

A multi-center registry can provide robust real-world evidence regarding CMR diagnostic effectiveness and patient risk stratification, when imaging protocols, data collection, and reporting were standardized. Such evidence from the use of vasodilating stress CMR perfusion is currently limited.

## Methods

In 2006, we developed a web-based multicenter registry (CMR-Cooperative, CMRCOOP) specific for performance of clinical CMR. This registry aimed to standardize imaging protocol, collection of clinical data, data interpretation, and reporting. All patient identifying information was encrypted. All data was stored and protected by intranet servers and site-specific administrative access. We identified patients who were referred for vasodilating CMR studies with suspected ischemia from 3 major CMR and 1 European centers. Presence of > 1 segment of abnormal stress perfusion without LGE defines ischemia presence and the number of ischemic segments defines ischemia extent. Major hard outcomes (MACE) including all-cause mortality and acute MI were assessed and were associated with CMR evidence of ischemia, using Cox regression.

## Results

**Figure 1:**
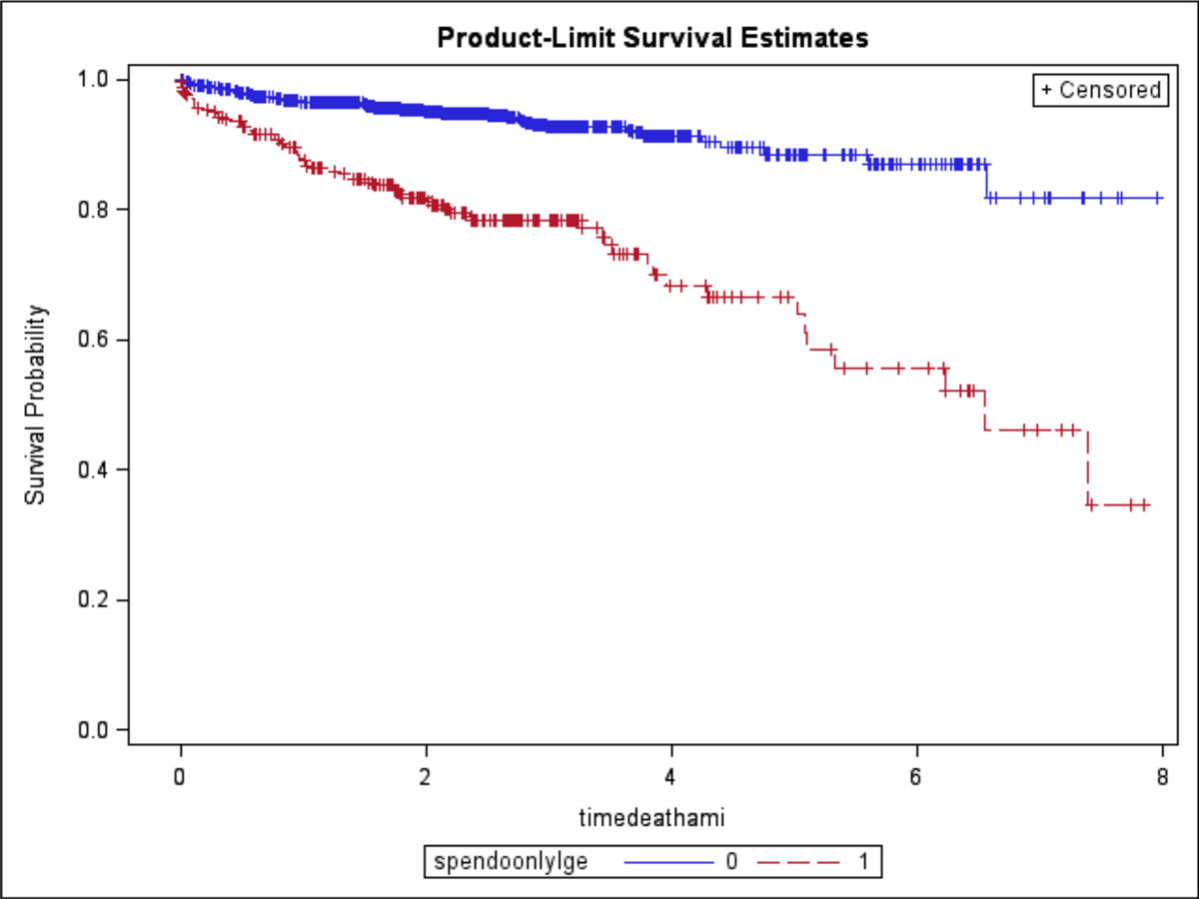


**Figure 2:**
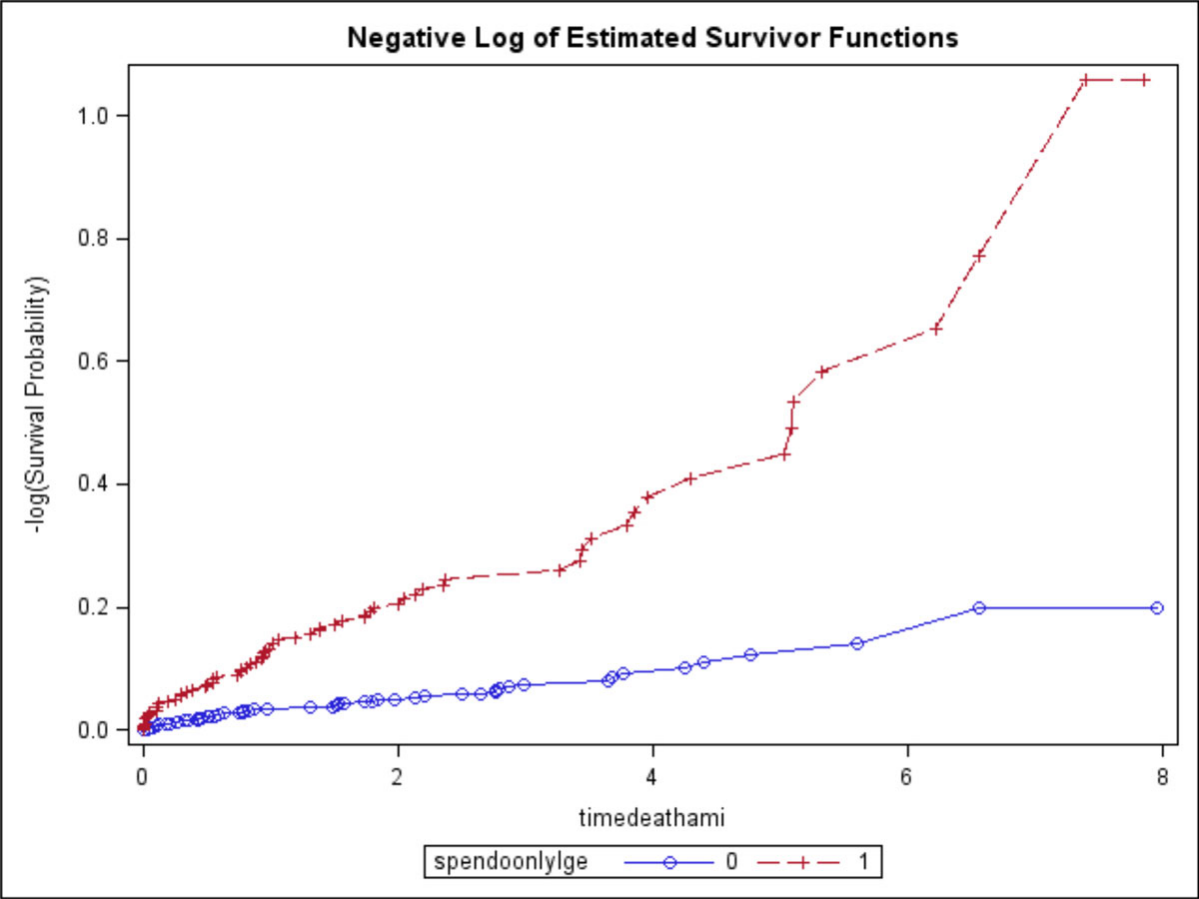


From the 4 centers, 1729 patients were followed for MACE. At a median of 2.3 years, 146 patients (8%) experienced MACE (80 deaths, 66 acute MIs). Ischemia presence and ischemia extent both demonstrated strong association with MACE: Ischemia presence portended to a near 4-fold increase in MACE (P < 0.0001); whereas for every segmental increase of ischemia extent, hazards of MACE increased by 14% (P < 0.0001). Kaplan-Meier survival curves are shown (Figure 1). Cumulative survival curves (Figure 2) demonstrated that vasodilating CMR appeared to have a high negative event rate for the initial 4 years after the CMR study.

## Conclusions

From this multicenter web-based registry with standardized imaging and data-collection methods, vasodilating CMR demonstrates robust patient risk stratification in patients with suspected ischemia.

## Funding

No external funding.

